# Diagnostic Value of Ultrasound for Sternal Fractures in Patients with Trauma Experiencing Anterior Chest Wall Pain

**DOI:** 10.3390/jcm13175123

**Published:** 2024-08-29

**Authors:** Hoonsung Park, Maru Kim, Dae-Sang Lee, Tae Hwa Hong, Doo-Hun Kim, Hangjoo Cho

**Affiliations:** 1 Division of Acute Care Surgery, Department of Surgery, Korea University Anam Hospital, Seoul 02841, Republic of Korea; niedwynn@naver.com; 2 Department of Trauma Surgery, Uijeongbu St. Mary’s Hospital, College of Medicine, The Catholic University of Korea, Seoul 06591, Republic of Korea; maru@catholic.ac.kr (M.K.); copymed@naver.com (D.-S.L.); spartan9717@gmail.com (T.H.H.); 3 Department of Surgery, Armed Forces Capital Hospital, Seongnam 13574, Republic of Korea; kgb1cia2@naver.com

**Keywords:** ultrasonography, sternum, thoracic injuries, CT scan, radioisotope scanning

## Abstract

**Background:** Ultrasound is an attractive modality for the confirmation of sternal fractures in patients with trauma because of its easy, quick, and accurate nature, as well as its increased availability for focused assessment with sonography for trauma at the bedside. We aimed to confirm the diagnostic value of ultrasonography for sternal fractures in patients with trauma, anterior chest wall pain, and tenderness. **Methods:** This retrospective observational study included patients visiting a single regional trauma center from March 2022 to February 2023, diagnosed with sternal fractures via chest CT and bone scans, who underwent sternal ultrasound. **Results:** Twenty-six patients were divided into two groups: those with sternal fractures diagnosed with an initial chest CT scan (*n* = 19) and those without fractures (*n* = 7). Using ultrasound, 23 patients (88.5%) were diagnosed with sternal fractures. In the initial CT scan (+) group, all 19 patients (100%) were diagnosed using ultrasound. In the initial CT scan (−) group, four (57.1%) of the seven patients were diagnosed using ultrasound. In the initial CT scan (+) group, 14 (73.7%) of the 19 patients underwent bone scans and all 14/14 (100%) were diagnosed with sternal fractures. In the initial CT scan (−) group, seven (100%) patients underwent bone scans, and all were diagnosed with sternal fractures. **Conclusions:** Ultrasound is useful for the diagnosis of sternal fractures, with sensitivity of 88.5%. Therefore, in patients with blunt trauma experiencing anterior chest wall pain and tenderness, sternal ultrasonography might be helpful in diagnosing sternal fractures as an adjunct to chest CT and bone scans.

## 1. Introduction

Anterior chest wall trauma and blunt and deceleration injuries are the most common causes of sternal fractures. The reported incidence of sternal fractures in motor vehicle collisions ranges from 3 to 6.8% [1,2]. In particular, an obvious increase has been observed in the incidence of accidents since the introduction of seatbelts [3,4]. Most sternal fractures involve the body of the sternum, although they can occur anywhere on the sternum. Sternal fractures most commonly occur in the top third of the sternal body [5,6]. Sternal fractures are typically associated with pain in the anterior chest wall. The most prevalent symptom of sternal fractures is point tenderness over the sternum [7].

Patients with suspected sternal injuries are typically evaluated using chest radiography. However, the sensitivity of anteroposterior radiography in identifying sternal fractures is only 50% [7]. Because most sternal fractures are transverse and any displacement can develop in the sagittal plane, a lateral view can enhance the sensitivity of diagnosis [8,9]. However, obtaining lateral chest radiographs in patients with trauma can be challenging, particularly in cases with multiple injuries [10]. Computed tomography (CT) has emerged as the gold standard [11]. However, CT scans require considerable examination time. Thus, evaluating patients with instability using CT scans is not appropriate [12]. Bone scans can also be used as adjuncts to diagnose fractures. Patients with multiple traumatic injuries can benefit from this screening method that identifies missing skeletal injuries [13]. In a study that conducted bone scans in chest trauma patients, the sensitivity and specificity of bone scans for fractures of the thorax were 99.4% and 90.4%, respectively [14]. In a systematic review, not in the chest but in scaphoid fractures, the sensitivity, specificity, positive predictive value, and negative predictive value of bone scans were 92.8%, 90.9%, 72.2%, and 99.2%, respectively [15].

Focused assessment with sonography for trauma (FAST) and extended focused assessment with sonography for trauma (EFAST) are commonly used for the initial evaluation of patients with trauma. Ultrasound has emerged as a highly attractive and preferred option for the confirmation or ruling out of sternal fractures in patients with trauma. This preference is due to ultrasound’s ability to provide an easy, rapid, and highly accurate evaluation of the sternum. Additionally, the growing availability of ultrasound for FAST and EFAST at the bedside, whether in the emergency room or trauma bay, has further solidified its role in the prompt and effective diagnosis of traumatic injuries [10]. However, studies on the use of ultrasonography for sternal fractures, especially in patients with trauma, are limited.

Treatment for isolated and stable sternal fractures is conservative management with adequate analgesia. However, for unstable or significantly displaced sternal fractures, surgical fixation may be indicated [7]. Significant displacement is usually identified in CT scans. However, for minimal displacement that is difficult to identify in CT scans, ultrasound may be helpful for the diagnosis of sternal fractures and the start of adequate pain control. Sternal fractures are associated with prolonged pain and physical limitations for up to 28 weeks [16]. In addition, if sternal fractures can be identified with EFAST before CT scans, this may be helpful for the management of patients as it may indicate a possible cardiac injury.

Among patients who initially visit a hospital, those who complain of anterior chest wall pain can be easily examined via sternal ultrasound followed by EFAST. This diagnostic process is notably efficient, often requiring only a few seconds to complete, making it an invaluable tool in the timely evaluation and management of these patients. Therefore, this study aimed to confirm the diagnostic value of ultrasound for sternal fractures in patients with trauma, anterior chest wall pain, and tenderness.

## 2. Materials and Methods

**Figure 1:**
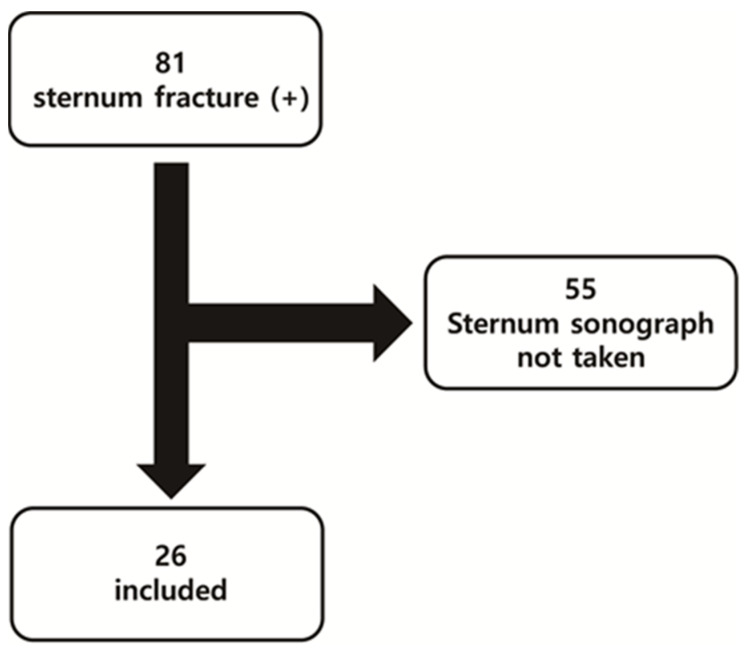
Flow chart showing the selection of the study subjects.

This retrospective observational study included patients who visited a single regional trauma center between March 2022 and February 2023 and were diagnosed with sternal fractures. The final diagnosis of sternal fracture was obtained using chest CT and bone scans. In this study, the gold standard for the diagnosis of sternal fractures was the chest CT scan, and a bone scan served as an adjunct for the diagnosis. This study involved a total of 81 patients with sternal fractures, of which 55 were excluded because sternal ultrasound was not performed for any reason. Ultimately, 26 patients were included in this study (Figure 1).

**Figure 2:**
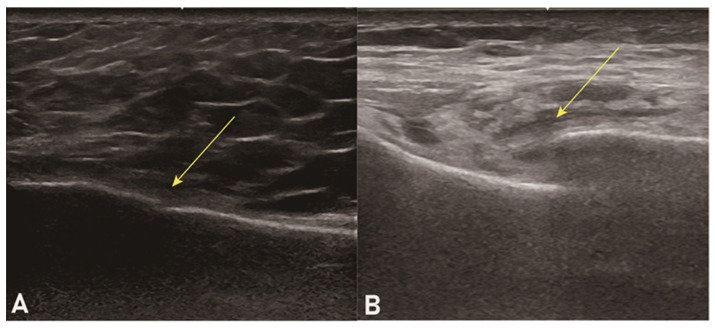
Two example images of sternal ultrasound showing positive findings with loss of continuity in the sternal cortex (arrows) ((**A**): minimal displacement, (**B**): moderate displacement).

Sternal ultrasound was performed by two trauma surgeons skilled in sonography, whose experience with ultrasound examination amounted to 6 and 7 years, respectively. The indication for sternal ultrasound was determined when the patient complained of anterior chest wall pain at the time of the visit, with positive tenderness. It was implemented following EFAST. The sternum was evaluated longitudinally using a linear probe. The diagnosis of a sternal fracture was obtained when a loss of continuity in the sternal cortex was observed (Figure 2) and the location of the maximum point of tenderness on the probe coincided with the location of the loss of continuity in the sternal cortex.

A chest CT scan was performed for all patients who complained of chest wall pain. A bone scan was performed between 7 and 14 days after the injury, unless the patient refused the examination. Sternal fractures were divided into the manubrium and body, according to their location. The body was divided into three parts for the purpose of fracture identification: upper, middle, and lower. Diagnostic radiologists were responsible for the primary interpretation and reporting of chest CT scans. Doctors of the Nuclear Medicine Department were responsible for reporting the bone scans.

Statistical analysis was performed using the R package ‘GGally 2.1.2’ and ‘moonBook 0.3.1’. Fisher’s exact test was used to analyze the sex, mechanism, mechanism details, ultrasound, follow-up CT scan, follow-up CT scan (+), bone scan, bone scan (+), and fracture location. Student’s *t*-test was used to analyze the age, injury severity score (ISS), CT follow-up day, and bone scan day. The Mann–Whitney U test was employed to analyze the body mass index (BMI).

This study was approved by the Institutional Review Board of the hospital to which the authors belonged (UC23RISI0140), which waived the requirement for informed consent.

## 3. Results

The patient characteristics are shown in Table 1. Sternal fractures were diagnosed in all 26 patients. The patients were divided into two groups according to the diagnosis of the sternal fracture on the initial chest CT scan. No differences in sex, age, BMI, the mechanism of injury, the mechanism details, or ISS were observed between the two groups. Of the entire patient group, 16 (61.5%) patients were female. The mean age of all patients was 56.8 years, ranging from 25 to 78 years. The median BMI was 25.8. Regarding the mechanism of injury, car traffic accidents (TAs) accounted for the highest number of cases (22). Regarding the mechanism details, the seat of the driver accounted for 17 (65.4%) cases. The mean ISS was 12.2.

A comparison of the sternal fracture diagnoses using a CT scan, ultrasound, and bone scan is shown in Table 2. Finally, the 26 patients with sternal fractures were divided into two groups: those with sternal fractures on the initial CT scan (*n* = 19) and those without sternal fractures (*n* = 7). Using ultrasound, 23 (88.5%) of the 26 patients were diagnosed with sternal fractures, indicating that the sensitivity of ultrasound for the diagnosis of sternal fractures was 88.5%. In the initial CT scan (+) group, all 19 patients (100%) were diagnosed using ultrasound. In the initial CT scan (−) group, four (57.1%) of the seven patients were diagnosed using ultrasound. In the initial CT scan (−) group, patients who were not diagnosed with sternal fractures by ultrasound (*n* = 3) underwent follow-up CT scans, and all three (100%) were diagnosed with sternal fractures.

In the initial CT scan (+) group, 14 (73.7%) of the 19 patients underwent bone scans, and all 14 (100%) were diagnosed with sternal fractures. In the initial CT scan (−) group, all seven patients (100%) underwent a bone scan, and all of them were diagnosed with sternal fractures.

The mean CT follow-up time was 41.3 days for the initial CT scan (−) group, which was significantly longer than that (12.0 days) for the initial CT scan (+) group (*p* < 0.01). The mean bone scan time was 14.7 days in the initial CT scan (−) group and 12.4 days in the initial CT scan (+) group, showing no significant difference between the two groups.

No significant differences in the fracture location were observed between the two groups. They comprised the mid-body (*n* = 11, 42.3%), upper body (*n* = 7, 27%), manubrium (*n* = 5, 19.2%), and lower body (*n* = 3, 11.5%).

The detailed data of all 26 patients enrolled in this study according to the diagnostic modalities are presented in Table 3. In the initial chest CT scan (−) group (*n* = 7), four of seven were occult fractures and three of seven were missed fractures.

## 4. Discussion

The most critical limitation of the present study was that we could not analyze the specificity and positive or negative predictive values of the diagnostic modalities. This limitation arose from the structure of the study. The only diagnostic performance measure available for assessment was the sensitivity of ultrasound, which was found to be 88.5%. The sensitivity of 88.5% in this study was comparable to the 91% sensitivity obtained during a meta-analysis conducted by Yousefifard et al. [11]. In the present study, we conducted ultrasound when the patient complained of tenderness and anterior chest wall pain at the time of the visit. Therefore, no negative control group for the fractures was present. According to the structure of this study, statistical analyses of ultrasound (+) and bone scans (+) were not performed because they did not show absolute diagnostic accuracy but relative accuracy. The results obtained from this study showed that sternal ultrasound and bone scans could be used to diagnose sternal fractures not detected by the initial chest CT. 

Moreover, a total of 55 patients were diagnosed with sternal fractures without undergoing sternal ultrasound. This was due to various clinical reasons: the patients were unstable, had severe fractures, or were experiencing a compromised mental status that precluded the use of ultrasound. These conditions made it impractical or unsafe to perform the ultrasound examination. The mean ISS of this group was 17.2 ± 9.1, which was significantly higher than the ISS (12.2 ± 7.8) of the included patients (*p* = 0.01). In the case of patients with severe trauma with an unstable status, performing sternal ultrasound following EFAST was difficult, and it could also have harmed the patients because of the time consumption, even if it takes seconds. This study was conducted at a regional trauma center in the Republic of Korea that functioned as a Level 1 trauma center. Therefore, enrolling patients in a prospective study and implementing sternal ultrasound for all visiting patients to establish a control group was difficult.

No consensus has been reached upon on the gold standard for the diagnosis of sternal fractures [7], but chest radiography, chest CT scans, ultrasound, and bone scans can be used. Chest radiography was previously considered the gold standard. Currently, a chest CT scan is considered the gold standard [17]. Depending on the status and condition of the patient during a visit, many diagnostic modalities can be chosen. Therefore, determining a gold standard for the diagnosis of sternal fractures is difficult.

The mean CT follow-up time (mean) in the group without sternal fractures on the initial chest CT scan was 41.3 days, which was significantly longer than that (12.0 days) in the group with sternal fractures on the initial chest CT scan (*p* < 0.01). Patients who were initially found to have no sternal fractures on the initial chest CT scan generally experienced a relatively short length of hospital stay. Consequently, follow-up CT scans were not conducted during their hospitalization. Instead, these follow-up scans were scheduled to be performed on an outpatient basis after discharge. As a result, there was potential for delays in diagnosing the sternal fractures. This delay could be attributed to the fact that the follow-up CT scans to identify or confirm the fractures were not performed while the patients were still in the hospital, potentially postponing their diagnosis until after their discharge.

Among the seven patients who had an initially negative chest CT scan result, three were not confirmed to have sternal fractures by ultrasound. Patients were diagnosed with sternal fractures on follow-up CT and bone scans. Two out of three cases were occult fractures and one out of three was a missed fracture. The missed fracture was located on the posterior side of the sternum, which is difficult to detect using ultrasound because of posterior acoustic shadowing. The difficulty in evaluating the sternal posterior side has been described previously [10,18]. Thus, a prospective randomized study including a control group is needed to evaluate the accuracy of sternal ultrasonography in the future.

**Figure 3:**
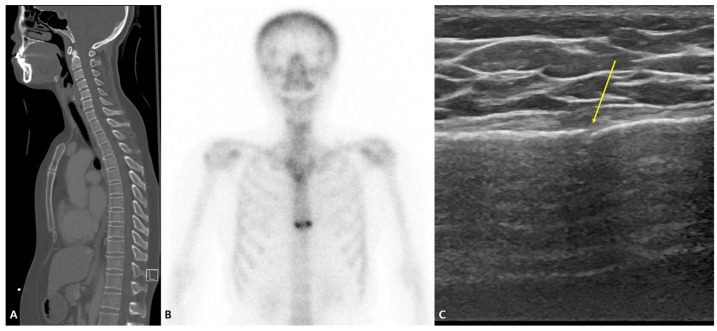
Three images of a case (number 22 in Table 3) with the (**A**) initial chest CT scan (−), (**B**) bone scan (+), and (**C**) ultrasound (+). Loss of continuity in the sternal cortex (arrow).

A bone scan was performed in all patients in the initial chest CT (−) group (*n* = 7), and all seven patients were found to have sternal fractures via the bone scan. Figure 3 shows an example of an initial chest CT scan (−), bone scan (+), and ultrasound (+). Regarding bone scans in diagnosing fractures, they are highly sensitive in detecting fractures; however, they can be nonspecific. When combined with clinical findings, bone scans may be more accurate than other imaging modalities. However, in clinical practice, this can cause patients to undergo unnecessary procedures or treatments. Furthermore, a bone scan is an invasive technique believed to be inappropriate for use in some populations, especially children [19].

In this study, occult fractures were defined as no fracture signs revealed on the initial chest CT scan. Dating back to the 1970s, there have been reports of the use of bone scans to diagnose occult fractures [20,21]. In a meta-analysis, the sensitivity and specificity of bone scans for the diagnosis of occult hip fractures were 87% and 96%, respectively [22]. In patients with anterior chest wall pain after blunt trauma, if chest radiography and chest CT scans fail to detect an injury, further diagnostic evaluations, such as bone scans, should be considered to identify occult sternal fractures [23].

## 5. Conclusions

Ultrasound is useful for the diagnosis of sternal fractures, with sensitivity of 88.5%. Therefore, in patients with blunt trauma with anterior chest wall pain and tenderness, sternal ultrasound might be helpful in diagnosing sternal fractures as an adjunct to chest CT and bone scans.

## Figures and Tables

**Table 1:** Characteristics of patients analyzed in this study.

	Fracture (+) in the Initial CT Scan (*n* = 19)	Fracture (−) in the Initial CT Scan (*n* = 7)	Total (*n* = 26)	*p*-Value
Sex				0.19
Male	9 (47.4%)	1 (14.3%)	10 (38.5%)	
Female	10 (52.6%)	6 (85.7%)	16 (61.5%)	
Age (mean)	57.3 ± 11.7	55.6 ± 19.8	56.8 ± 13.9	0.78
BMI (median)	24.5 ± 3.1	29.3 ± 10.0	25.8 ± 6.0	0.26
Mechanism				0.40
Motorcycle TA	0 (0.0%)	1 (14.3%)	1 (3.8%)	
In car TA	17 (89.5%)	5 (71.4%)	22 (84.6%)	
Others	2 (10.5%)	1 (14.3%)	3 (11.5%)	
Mechanism details				0.81
Passenger seat	3 (15.8%)	1 (14.3%)	4 (15.4%)	
Driver seat	13 (68.4%)	4 (57.1%)	17 (65.4%)	
Others	3 (15.8%)	2 (28.6%)	5 (19.2%)	
Injury severity score (mean)	13.5 ± 7.7	8.7 ± 7.5	12.2 ± 7.8	0.17

CT: computed tomography; BMI: body mass index; TA: traffic accident.

**Table 2:** Comparison of sternal fracture diagnosis using CT scan, ultrasound, and bone scan.

	Initial Chest CT Scan (+) (*n* = 19)	Initial Chest CT Scan (−) (*n* = 7)	*p*-Value
Ultrasound taken	19 (100%)	7 (100%)	1
Ultrasound (+)	19 (100%)	4 (57.1%)	N/A
Follow-up CT scan taken	8 (42.1%)	5 (71.4%)	0.18
Follow-up CT scan (+)	8 (100%)	5 (100%)	1
Bone scan taken	14 (73.7%)	7 (100%)	0.34
Bone scan (+)	14 (100%)	7 (100%)	N/A
CT follow-up day (mean)	12.0 ± 10.3	41.3 ± 13.6	<0.01
Bone scan day (mean)	12.4 ± 9.4	14.7 ± 8.7	0.62
Fracture location			0.57
Manubrium	3 (15.8%)	2 (28.6%)	
Upper	6 (31.6%)	1 (14.3%)	
Mid	7 (36.8%)	4 (57.1%)	
Lower	3 (15.8%)	0	

CT: computed tomography.

**Table 3:** Results of diagnosis by modality.

Number	Initial Chest CT Scan	Ultrasound	Occult or Missed Fracture ^(a)^	Follow-Up Chest CT Scan	Bone Scan
1	+	+		+	+
2	+	+		+	+
3	+	+		+	+
4	+	+		+	+
5	+	+		+	+
6	+	+		+	+
7	+	+		+	N/A
8	+	+		+	N/A
9	+	+		N/A	N/A
10	+	+		N/A	N/A
11	+	+		N/A	N/A
12	+	+		N/A	+
13	+	+		N/A	+
14	+	+		N/A	+
15	+	+		N/A	+
16	+	+		N/A	+
17	+	+		N/A	+
18	+	+		N/A	+
19	+	+		N/A	+
20	−	+	occult	+	+
21	−	+	occult	+	+
22	−	+	missed	N/A	+
23	−	+	missed	N/A	+
24	−	−	missed	+	+
25	−	−	occult	+	+
26	−	−	occult	+	+

+: sternal fracture positive; −: sternal fracture negative; N/A: not assessed; occult fractures: no fracture signs revealed on the initial chest CT scan; missed fractures: diagnosis was not obtained via the initial chest CT scan. ^(a)^ Initial chest CT scans (−) were reviewed by trauma surgeons when sternal fractures were diagnosed using other modalities.

## Data Availability

Data supporting the findings of this study are available from the corresponding author upon request.
